# How effective is the health promotion policy in Sichuan, China: based on the PMC-Index model and field evaluation

**DOI:** 10.1186/s12889-022-14860-9

**Published:** 2022-12-20

**Authors:** Yanlin Yang, Jing Tang, Zhixin Li, Jin Wen

**Affiliations:** 1 Institute of Hospital Management, West China Hospital, Sichuan University, Chengdu, 610041 China; 2 Sichuan Center for Disease Control and Prevention, Chengdu, 610041 China

**Keywords:** Health promotion policy, PMC-Index model, Expert field evaluations, Correlation analysis

## Abstract

**Background:**

Many countries around the world highlight the health in all policies (HiAP). However, most of the related research focused on the influential factors and implementation strategies, with less concern on the evaluation of HiAP. In response to HiAP's call, the Chinese government has proposed health promotion policies (HPPs) in counties or districts, the evaluation of HPPs in sample counties or districts of Sichuan province in China is an essential basis for optimizing policy content, improving policy implementation, and ensuring health promotion's continuous and efficient operation.

**Methods:**

This paper established an evaluation system for HPPs based on the PMC-Index model and then quantitatively analyzed 37 representative HPPs from the pilot areas in Sichuan province. In addition, a team of experts conducted a field assessment.

**Results:**

The results showed that the average PMC index of 37 HPPs was 7.091, and correlation analysis showed that there was a significant correlation between the PMC index and expert score.

**Conclusions:**

This study indicates that the overall consistency of HPPs was good and proves a connection between the formulation and implementation of HPPs.

## Background

The Health in all Policies (HiAP) was proposed in 2013. According to the World Health Organization, to improve the population's health and promote health equity, the HiAP emphasizes the cross-sectoral search for synergies, the integration of health outcomes, and the avoidance of harmful health effects [1]. Now, HiAP is a term used to describe efforts to improve health by incorporating health considerations into decision-making across policy domains [2]. HiAP has become one of the basic guidelines in China's health career in the same year, which means that health has been highly prioritized in the development of public policy and incorporated into all stages of policy-making [3].

With an aging population, a growing health burden, an outbreak of infections, and increasing health inequalities, a switch from disease treatment-oriented to prevention-oriented health policy-making could help address the prevalent health challenge [4]. The world's health focus is shifting from disease-specific treatment to universal, whole-cycle health[5]. However, it is essential to point out that the health policies shift still localized in unique domain-specific, such as prevention and treatment of chronic diseases, management of mental health during the epidemic, the control of childhood obesity, etc [6]. There remains a lack of systematic understanding of the health policy reform. The intricate and complex relations among the health system elements further affirm the need for a systematic approach to tackling health system challenges. HiAP is a practical and valuable tool to conduct system governance.

Through the review over HiAP related research at home and abroad, three themes are summarized by analyzing documents: the conceptualization of HiAP, the adoption of HiAP, and the implementation of HiAP [7]. Firstly, In terms of the conceptualization of HiAP, which is conceptualized as a method, strategy or framework. For example, Yuan Yanfei disseminated and popularized the concept, development history, action framework and international experience of HiAP [8]. Peters highlighted the cross-sectoral collaboration of HiAP [9]. Secondly, in terms of the adoption of HiAP, the main focus is on the influencing factors. For example, Pintodiscussed the impact of economic factors on the implementation of HiAP [10]. Hofstad pointed out the impact of Norwegian bureaucracy on HiAP [11], and Zeeb advocated the consideration and strengthening of environmental themes in HiAP [12]. Thirdly, in terms of the implementation of HiAP, Newman applied HiAP strategy to obesity [13]. Qichao Chen applied HiAP to hospital management research [14], and Vassiliou advocated the application of HiAP to healthcare policy [15]. At present, the related concepts, strategies and measures of HiAP at home and abroad are relatively clearly clarified in different perspectives. Some developed countries have designed HiAP operating mechanisms according to their national conditions, such as the interdepartmental cooperation committee system in the Netherlands [16] and the HiAP working Group in Boston, USA [17]. However, domestic studies on HiAP are mostly concentrated in a single field, especially in the field of health, showing a narrow fragmented state of research. It must be emphasized that HiAP is a complex multi-factorial, multistage process, which gives priority to well understanding the precise mechanisms. And there's very little research about evaluations of HiAP, including using the scoping review approach to analyze the policy texts [7]. Therefore, the traditional single-method evaluations should be transferred to a comprehensive and reliable evaluation [18].

Universal health coverage is a global priority, effectively helping reduce the disease burden and promote sustainable development [19]. With a large population, it is strategically important for China to improve its national health [20]. In 2014, the Chinese government actively responded to the concept of HiAP, and launched the pilot project of HPPs nationwide that year. The project has become an important way to implement the HiAP strategy construction and realize the health of the whole people. In 2017, HPPs was fully implemented nationwide, and all sectors of society responded positively and participated fully.

This study focuses on the HPPs in Sichuan province, which is vast in size (486,000 km2) and densely populated (83.41 million), proper for being the research subject [21]. For the moment, Sichuan province has selected 37 counties/districts as pilot projects to implement the HPPs. Each region has developed its own "Regional Health Promotion Plan" based on the basic national policy. In 2017, the Sichuan Provincial Health and Family Planning Commission built a big health environment to integrate health into all policies. In 2018, Leshan City in Sichuan Province promoted health concepts to the public by introducing mass media. In 2019, Yaan City in Sichuan Province launched a health promotion site. In 2020, the People's Government of Sichuan Province made efforts to safeguard life-cycle health. In 2021, the Sichuan Health Action Promotion Committee standardized management and strengthened the monitoring and evaluation of the Healthy China Action. The HPPs in Sichuan Province has experienced from identification to promotion and deepening, and then to supervision and testing. Therefore, the next most important concern is the evaluation of HPPs in Sichuan Province, taking factors related into consideration, including whether HPPs is more consistent with similar policies? What is HPPs's dilemma in practice? What is next for improvement? Through the evaluation of HPPs in Sichuan, we can find out the problem more comprehensively, summarize the experience and form better policy pathways.

Policy evaluation is a very crucial part of the analysis of public policy [22]. The results can provide a basis for rational allocation of health resources and improvement of health policy planning. In the past, the government often introduced expert assessment as an essential means of policy assessment, but it is prone to subjective bias [23]. China's HPPs still lacks a comprehensive, easy-to-operate, scientific evaluation method of the pros and cons of such policies. A variety of approaches on policy evaluation has been adopted, but the more current cutting-edge approach is the PMC-index model.

To assess the strengths and weaknesses of policies at multiple levels, Ruiz Estrada presented the Policy Model Coherence Index in 2011 [24]. The PMC-Index model does not limit the number of secondary indicators included in evaluations [25], while visually presenting the advantages and disadvantages of policies and the level of internal consistency of policies in the form of curved graph. In this study, words extracted from the policy text, such as health, promotion, environment, service, etc., were used as criteria to further the PMC-Index model so as to be properly applied to our research. In recent years, the PMC-Index model has become more widely used and has become a popular way to evaluate policies. In the field of industrial economy, Hu Feng et al. and Xu Liying et al. respectively applied the PMC-Index model to China's robot industry and biomedical industry [26, 27]. In the environmental field, Dai et al. used the PMC-index model to evaluate the effectiveness of implementing green development policies in the Yangtze River Economic Belt of China [25]. Liu Yating et al. applied the PMC index model to study China's long-term and water pollution prevention and control policies [28]. In the field of administrative management, Zhou Haiwei et al. applied the PMC-Index model to reservoir migration policy, which has strong practical application value [29].

In conclusion, compared with other evaluation methods, PMC-Index model combines quantitative and qualitative methods, which is more comprehensive, objective and intuitive. Moreover, each district and county government in Sichuan Province continuously expands and improves regional health policies based on HiAP, which provides a reference template for the evaluation of policy consistency. Therefore, this study combined PMC index model and expert field evaluation to evaluate the consistency of HPPs in Sichuan Province and explore the correlation between policy formulation and implementation, to analyze the advantages and disadvantages of HPPs in Sichuan, and provide theoretical reference for policy optimization and innovation.

## Methods

### PMC evaluation

In this study, HPPs was quantified using the PMC-Index model, consisting of four steps. Firstly, we reviewed and summarized 52 key policy articles based on the theme and content of the research. Then text and co-word matrices were established by analyzing and selecting high-frequency words using ROSTCM 6.0 software. Secondly, based on the results of text mining and literature review, the primary variable was designed to meet the research needs, the secondary variable was expanded, and the evaluation system was established. Thirdly, we selected 37 HPPs from each pilot for analysis. By text analysis, the binary count method was used to assign values to the secondary variables, and then a multi-input policy table was established. Finally, the 37 HPPs were rated based on the policy consistency criteria (see Table 1). The validity, advantages and disadvantages of policy texts were evaluated by constructing three-dimensional matrices and painting a PMC surface.

**Table 1:** Policy consistency rating sheet

**Code**	**9~10**	**7~8.99**	**5~6.99**	**0~4.99**
Connotation	Excellent consistency	Good consistency	Acceptable consistency	Poor consistency
Gade	A	B	C	D

#### Data sources and research samples

The search of policies was carried out using the following keywords related: health in all policies, health promotion policies, and prefectural health promotion plan. We retrieved policies from the Chinese government website, the official website of the National Health Commission of China, the website named magic weapon of Peking University, and the official website of pilots. The retrieval dates were from January 1, 2013, to May 30, 2022. We have reviewed several types of policy documents, such as programs, regulations, observations, measures, plans, guidelines, etc., with the focus on those with specific action plans. After initial screening, 52 documents were obtained, including 15 national-level and provincial-level policies and 37 district-level or county-level policies from 37 pilot regions. These samples were identified as P1 to P52, and policy text excerpts are presented in Table 2.

**Table 2:** Policy Texts Summary

**Code**	**Policy Text Name**	**Issuing Agency**	**Date**
P1	Notice of the national health and Family Planning Commission on printing and distributing the action plan for the promotion of the health literacy of the whole people (2014-2020)	the National Health and Family Planning Commission	May 9, 2014
P2	Guidance on strengthening health promotion and education	Propaganda Department of National Health Commission of the People's Republic of China	November 18, 2016
P3	Notice of the general office of the family health and Family Planning Commission on the announcement of the first batch of national health promotion county (District) project pilot	the National Health and Family Planning Commission	March 29, 2017
P4	Circular on the issuance of the plan of action for a healthy lifestyle for all (2017-2025)	the National Health and Family Planning Commission; General office of the general administration of sport; General office of the national federation of trade unions; General Office of All-China Women's Federation, etc	April 27, 2017
P5	Health and Family Planning Commission Press Conference on Health Promotion County (District) Construction	the National Health and Family Planning Commission	January 25, 2018
P6	Healthy china action(2019–2030)	National planning development and information department	July 15, 2019
P7	Implementation Opinions of Sichuan Provincial People's Government on Promoting Healthy Sichuan Action	Sichuan provincial people's government	November 26, 2019
P8	Notice of the office of the health Sichuan action promotion committee on printing and distributing the workpoints of the health Sichuan action in 2021	Health Sichuan Campaign Committee Office	March 8, 2021
P9	Notice of patriotic health campaign committee, Sichuan province on printing and distributing the opinions of Sichuan province on the implementation of the patriotic health campaign	Health Sichuan Campaign Committee Office	October 28, 2021
P10	Notice of the general office of the national health and health commission on reporting the 2021 national health promotion county (district) technical evaluation and typical experience	the National Health and Family Planning Commission	February 16, 2022

We conducted word segmentation analysis and semantic network analysis on the above 52 policies by ROSTCM 6.0, and we obtained high-frequency words including "health," "education," "service," and "management".

Ultimately, we recognized 37 HPPs from pilot areas as the research object, and the quantitative policy evaluation was carried out by using the PMC-Index model.

#### Classification of Variables and Identification of parameters

For the comprehensive evaluation strategy, the following aspects were taken into consideration in selecting variables in this study. Ruiz Estrada [24] proposed 10 main variables, namely research types, research direction, data source, econometric methods adopted, study scope, research theoretical framework, policy modelling by sector, economic framework, geographical analysis, paper citations and 50 subvariables. On this basis, working with the evaluation of the existing policy, for example, Tingting Jia, Siqi Zhao would talk about policy nature, policy limitation, and public policy content [30, 31]. Yuying Zou, Yi Jihave mentioned policy incentives, such as policy areas, publishing institutions, policy evaluation [32, 33]. In addition, Wenjin Zhang also mentioned policy supervision, function, and Jinming Xing also mentioned policy perspective, the policy mix [34, 35]. Finally, based on the relevant policy texts of health promotion districts and counties in China and Sichuan Province, 10 first-level variables and 44 S-level variables were determined. The first-level variables are shown in Table 3, numbered X1-X10, namely policy nature and timeliness, policy relevance, incentives and constraints, policy subject and policy content, policy evaluation and issuing institutions, policy object and policy disclosure. Then we need to set parameters for each variable. Then we need to set parameters for each variable. Binary assignments were used to give equal importance and weight to all secondary variables. More specifically, if something in the policy text is consistent with the expression of the second variable, the assignment value is 1; conversely, the assignment value is 0 [36].

**Table 3:** Variables Setting of Quantitative Evaluation of HPPs

**Primary Variable**	**Secondary Variable**	**Two-Level Variable Evaluation Criteria**
X1 Policy nature	X1:1 prediction	Whether reflects the prediction? Yes is 1, no is 0
	X1:2 supervision	Whether reflects the supervision? Yes, 1, no, 0.
	X1:3 recommendation	Whether reflects the suggestion? Yes, 1, no, 0.
	X1:4 support	Whether reflects the support? Yes, it is 1, no, it is 0.
	X1:5 guidance	Whether reflects the guidance?Yes, 1, no, 0.
	X1:6 diagnosis	Whether reflects the diagnosis?Yes is 1, no is 0.
	X1:7 description	Whether reflects the description?Yes is 1, no is 0.
X2 Policy timeliness	X2:1 long term	Whether it involves content longer than 5 years? Yes is 1, no is 0.
	X2:2 medium-term	Whether it involves 3-5 years? Yes is 1, no is 0.
	X2:3 short term	Whether it involves 1-3 years? Yes, 1, no, 0.
	X2:4 temporary	Whether it involves content less than 1 year?Yes is 1, no is 0.
X3 Policy relevance	X3:1 national policy	Is it related to other national policies? Yes, 1; no, 0.
	X3:2 provincial policy	Is it related to other provincial policies? Yes, 1; no, 0.
	X3:3 municipal policy	Is it related to other municipal policies? Yes is 1, no is 0.
	X3:4 other policies	Is it related to other policies (county-level policies, etc.)? Yes, 1; no, 0.
X4 Incentives and constraints	X4:1 talent incentives	Whether is there talent incentive content? Yes is 1, no is 0.
	X4:2 fiscal incentives	Whether are there fiscal and tax incentives? Yes is 1, no is 0.
	X4:3 administrative approval incentives	Whether is there administrative approval support? Yes is 1, no is 0.
	X4:4 laws and regulations	Do laws and regulations support it? Yes is 1, no is 0.
X5 Policy subjects	X5:1 politics	Whether the policy involves the political field? Yes 1, no 0.
	X5:2 economy	Whether the policy involves the economic field? Yes is 1, no is 0.
	X5:3 technology	Whether the policy involves the technical field? Yes is 1, no is 0.
	X5:4 society	Whether the policy involves the social and people's livelihood? Yes, 1, no, 0.
	X5:5 environmental protection	Whether the policy involves the field of environmental protection? Yes is 1, no is 0.
X6 Policy content	X6:1 improve the policy system	Whether the content is to improve the policy system? Yes is 1, no is 0.
	X6:2 strengthen organizational management	Whether the content is to strengthen organizational management? Yes is 1, no is 0.
	X6:3 building a healthy place	Whether the content is to establish a healthy place? Yes is 1, no is 0.
	X6:4 spreading health culture	Whether the content is to spread healthy culture? Yes is 1, no is 0.
	X6:5 creating a healthy environment	Whether the content is to create a healthy environment? Yes is 1, no is 0.
	X6:6 foster healthy people	Whether the content is to cultivate healthy people? Yes is 1, no is 0
X7 Policy evaluation	X7:1 specific goals	Whether the policy has specific goals? Yes is 1, no is 0.
	X7:2 detailed planning	Whether the policy planning is detailed? Yes is 1, no is 0.
	X7:3 scientific Programme	Whether the policy programme is scientific? Yes is 1, no is 0.
	X7:4 sufficient basis	Whether the policy basis is sufficient? Yes is 1, no is 0
	X7:5 clear rights and responsibilities	Whether the policy has clear rights and responsibilities? Yes is 1, no is 0.
X8 Issuing agency	X8:1 people's congress	Whether the policy issuing agency is the people's congress? Yes is 1, no is 0.
	X8:2 the communist party of china(cpc) commission	Whether the policy issuing agency is the communist party of china(cpc) commission? Yes is 1, no is 0.
	X8:3 government offices	Whether the policy issuing agency is the local government office? Yes is 1, no is 0.
	X8:4 other government departments	Whether the policy issuing agency is other functional departments of the local government? Yes is 1, no is 0.
X9 Policy objects	X9:1 public institution	Whether it has an impact on institutions (schools, hospitals, etc.)? Yes is 1, no is 0.
	X9:2 enterprise units	Whether it affects the enterprise unit? Yes is 1, no is 0.
	X9:3 public environment	Whether it has an impact on the public environment (parks, trails, etc.)? Yes is 1, no is 0
	X9:4 communities	Whether it has an impact on the community? Yes is 1, no is 0.
	X9:5 families and individuals	Does it affect families and individuals? Yes is 1, no is 0.
X10 Policy disclosure	-	Whether the policy is open? Yes is 1, no is 0.

#### Building a multi-input-output table

To quantify the values of the main-variables, a multi-input-output table was designed at this section (see Table 4).

**Table 4:** Multi-Input-Output Table

**Primary variable**	**X1**	**X2**	**X3**	**X4**	**X5**	**X6**	**X7**	**X8**	**X9**	**X10**
Secondary variable	X1:1	X2:1	X3:1	X4:1	X5:1	X6:1	X7:1	X8:1	X9:1	-
	X1:2	X2:2	X3:2	X4:2	X5:2	X6:2	X7:2	X8:2	X9:2	
	X1:3	X2:3	X3:3	X4:3	X5:3	X6:3	X7:3	X8:3	X9:3	
	X1:4	X2:4	X3:4	X4:4	X5:4	X6:4	X7:4	X8:4	X9:4	
	X1:5				X5:5	X6:5	X7:5		X9:5	
	X1:6					X6:6				
	X1:7									

#### Measurement of the PMC-Index

There are usually four steps to calculate the PMC index. Firstly, a multi-input- output table was created to incorporate primary and secondary variables. Secondly, each secondary variable was assigned a value based on text analysis and formula (1) [2]. Thirdly, each primary variable was calculated according to formula (3). Fourthly-, the PMC index of policies was calculated by adding up the sum according to formula (4) [24, 37].

1 $$X\sim N[\mathrm{0,1}]$$

2 $$X=\{XR:[0:1]\}$$

3 $${X}_{i}(\sum_{j=1}^{n}\frac{{X}_{ij}}{T({X}_{ij})})$$

($i$ is recorded as primary variables, $i=1\dots n$;

$j$ is recorded as secondary variables, $j=1\dots m$;

${X}_{ij}$ is the score for $j$-th secondary variable in $i$-th primary variable;

$T({X}_{ij})$ is the number of secondary variables in $i$-th primary variable)

4 $$PMC=\sum_{i=1}^{n}{X}_{i}$$

($i$ is recorded as primary variables, $i=1\dots n$;

${X}_{i}$ is the score for $i$-th primary variable)

#### PMC-surface construction

PMC surfaces can visualize the policy's strengths and weaknesses. Since all policy documents are obtained from open websites, all policies receive a score of 1. Also, considering the symmetry and balance of the matrix, after removing P10, we created a matrix of size 3 * 3 to form a curved graph. The associated matrix is shown in formula (5) [24].

5 $$PMC-Surface=\left[\begin{array}{ccc}X1& X4& X7\\ X2& X5& X8\\ X3& X6& X9\end{array}\right]$$

### Expert field evaluations

The Health Promotion Policy Criteria (HPPsC) includes six primary indicators (organization and management, health policy, healthy places, healthy culture, healthy environment, healthy people) and 39 secondary indicators. The expert group scored 37 pilot areas at the field research site. The maximum HPPsC score was 1000, but the total score was transformed to a percentage grading system for a visual representation of the results, with a total score of 100. In terms of quality control, firstly, experts should be trained before field evaluation, so that each expert has the same understanding and standard for each evaluation item. Secondly, many evaluation items should be objectively existing data. Thirdly, each site has more than two experts to evaluate. If there is any inconsistency between the two experts, the third expert will evaluate until the consensus is reached.

### Correlation analysis

Correlations between the PMC-Index score and the field evaluation score by experts were estimated using the Person correlation coefficient by Spss26.0 software (IBM Corp). Significance tests were 2-tailed, with α=0.05.

## Results

### PMC evaluation

#### The PMC-Index of the policies

Access to existing research, the consultation of a policy's PMC index should be divided into four levels [25]. Scores range from 0 to 4.99 for poor consistency policies. Scores range from 5 to 6.99 for acceptable consistency policies. A score between 7 and 8.99 is a good consistency. A score between 9 and 10 is a perfect consistency for policy, and the more consistent the text, the better the overall policy guidance.

Then through text mining and content analysis, 37 policy texts of pilot areas were assigned, and the policy score was calculated according to the PMC index calculation formula. It is only P7 with perfect consistency. There are 17 policies with good consistency, namely P1, P5, P9, P10, P12, P17, P18, P20, P21, P23, P28, P29, P30, P31, P32, P34, and P37. There were 18 policies with acceptable consistency, P2, P3, P4, P6, P8, P11, P13, P14, P15, P16, P19, P22, P24, P25, P26, P27, P35, P36. One policy with poor consistency was P33. The average score of the PMC index is 7.091, which indicates that the policies of all districts and counties have good consistency (see Table 5). Among the scores of primary variables, the average score of X10 (policy disclosure) is the highest. The average score of X8 (issuing agency) is the lowest.

**Table 5:** PMC-Index

	**P1**	**P2**	**P3**	**P4**	**P5**	**P6**	**P7**	**P8**	**P9**	**P10**	**P11**	**P12**	**P13**	**P14**	**P15**	**P16**	**P17**	**P18**	**P19**
X1	0.857	0.857	0.714	0.857	1.000	0.429	1.000	0.857	1.000	0.429	0.571	0.714	0.857	0.857	0.714	1.000	0.714	1.000	0.714
X2	0.500	0.250	0.500	0.250	0.750	0.500	1.000	0.250	1.000	1.000	0.250	1.000	0.250	0.250	0.250	0.250	1.000	1.000	0.500
X3	0.500	0.750	0.250	0.250	0.750	0.250	1.000	0.500	0.500	0.750	0.500	1.000	0.250	0.500	0.250	0.250	0.750	0.750	0.000
X4	0.250	0.250	0.250	0.250	0.500	0.500	0.750	0.250	0.500	0.750	0.500	0.750	0.250	0.250	0.500	0.250	0.750	0.500	0.250
X5	0.800	0.800	0.800	0.600	1.000	0.600	1.000	0.800	1.000	0.600	1.000	0.800	0.800	0.800	0.600	0.800	0.600	1.000	0.600
X6	1.000	0.833	0.833	1.000	1.000	1.000	1.000	1.000	1.000	1.000	0.833	1.000	1.000	0.833	0.667	1.000	1.000	1.000	0.833
X7	1.000	0.800	1.000	1.000	1.000	1.000	1.000	1.000	1.000	1.000	0.600	1.000	0.800	0.600	0.600	0.800	1.000	1.000	0.800
X8	0.250	0.250	0.250	0.250	0.250	0.250	0.250	0.250	0.250	0.250	0.250	0.250	0.250	0.250	0.500	0.250	0.250	0.250	0.250
X9	1.000	0.600	0.600	1.000	1.000	1.000	1.000	1.000	1.000	1.000	1.000	1.000	1.000	1.000	0.600	1.000	1.000	1.000	1.000
X10	1.000	1.000	1.000	1.000	1.000	1.000	1.000	1.000	1.000	1.000	1.000	1.000	1.000	1.000	1.000	1.000	1.000	1.000	1.000
PMC	7.157	6.390	6.198	6.457	8.250	6.529	9.000	6.907	8.250	7.779	6.505	8.514	6.457	6.340	5.681	6.600	8.064	8.500	5.948
rank	B	C	C	C	B	C	A	C	B	B	C	B	C	C	C	C	B	B	C

	**P20**	**P21**	**P22**	**P23**	**P24**	**P25**	**P26**	**P27**	**P28**	**P29**	**P30**	**P31**	**P32**	**P33**	**P34**	**P35**	**P36**	**P37**	**Average of all**
X1	0.857	0.857	0.571	0.429	0.429	0.857	0.714	0.857	0.429	0.714	0.571	0.857	0.429	0.571	1.000	0.571	0.714	0.714	0.737
X2	1.000	0.500	0.250	1.000	0.500	0.500	0.500	0.500	0.750	0.750	1.000	0.500	1.000	0.250	0.500	0.500	0.500	0.500	0.588
X3	0.500	0.500	0.750	0.750	0.250	0.750	0.250	0.250	0.750	0.750	0.750	1.000	0.750	0.250	0.750	0.500	0.250	0.750	0.547
X4	0.500	0.500	0.250	0.750	0.500	0.000	0.000	0.250	0.500	0.250	0.500	0.500	0.750	0.000	0.750	0.750	0.000	0.250	0.412
X5	1.000	0.800	0.600	0.600	0.600	0.800	0.800	0.800	0.600	0.800	0.600	1.000	0.600	0.600	1.000	0.600	1.000	0.800	0.773
X6	1.000	1.000	1.000	1.000	1.000	1.000	1.000	1.000	1.000	1.000	1.000	1.000	1.000	0.500	1.000	1.000	1.000	1.000	0.955
X7	1.000	1.000	0.800	1.000	1.000	1.000	0.800	0.800	1.000	1.000	1.000	1.000	1.000	0.400	1.000	1.000	1.000	1.000	0.914
X8	0.250	0.250	0.250	0.250	0.250	0.250	0.250	0.250	0.250	0.250	0.250	0.250	0.250	0.250	0.250	0.250	0.250	0.250	0.257
X9	1.000	1.000	0.800	1.000	1.000	0.800	1.000	1.000	1.000	1.000	1.000	1.000	1.000	0.200	1.000	0.000	1.000	1.000	0.908
X10	1.000	1.000	1.000	1.000	1.000	1.000	1.000	1.000	1.000	1.000	1.000	1.000	1.000	1.000	1.000	1.000	1.000	1.000	1.000
PMC	8.107	7.407	6.271	7.779	6.529	6.957	6.314	6.707	7.279	7.514	7.671	8.107	7.779	4.021	8.250	6.171	6.714	7.264	7.091
rank	B	B	C	B	C	C	C	C	B	B	B	B	B	D	B	C	C	B	

#### The PMC-surface of the typical policies

The three-order square matrix was constructed with X1, X2, X3, X4, X5, X6, X7, X8, and X9. In this study, one policy sample (P7, P1, P3, P33) from each level of ABCD was selected to construct the PMC matrix and draw the surface map.

$$\begin{array}{c}P7=\left(\begin{array}{ccc}1& 0.75& 1\\ 1& 1& 0.25\\ 1& 1& 1\end{array}\right)\\ \begin{array}{c}P1=\left(\begin{array}{ccc}0.857& 0.25& 1\\ 0.5& 0.8& 0.25\\ 0.5& 1& 1\end{array}\right)\\ \begin{array}{c}P3=\left(\begin{array}{ccc}0.714& 0.25& 1\\ 0.5& 0.8& 0.25\\ 0.25& 0.833& 0.6\end{array}\right) \\ P33=\left(\begin{array}{ccc}0.571& 0& 0.4\\ 0.25& 0.6& 0.25\\ 0.25& 0.5& 0.2\end{array}\right)\end{array}\end{array}\end{array}$$

**Fig. 1:**
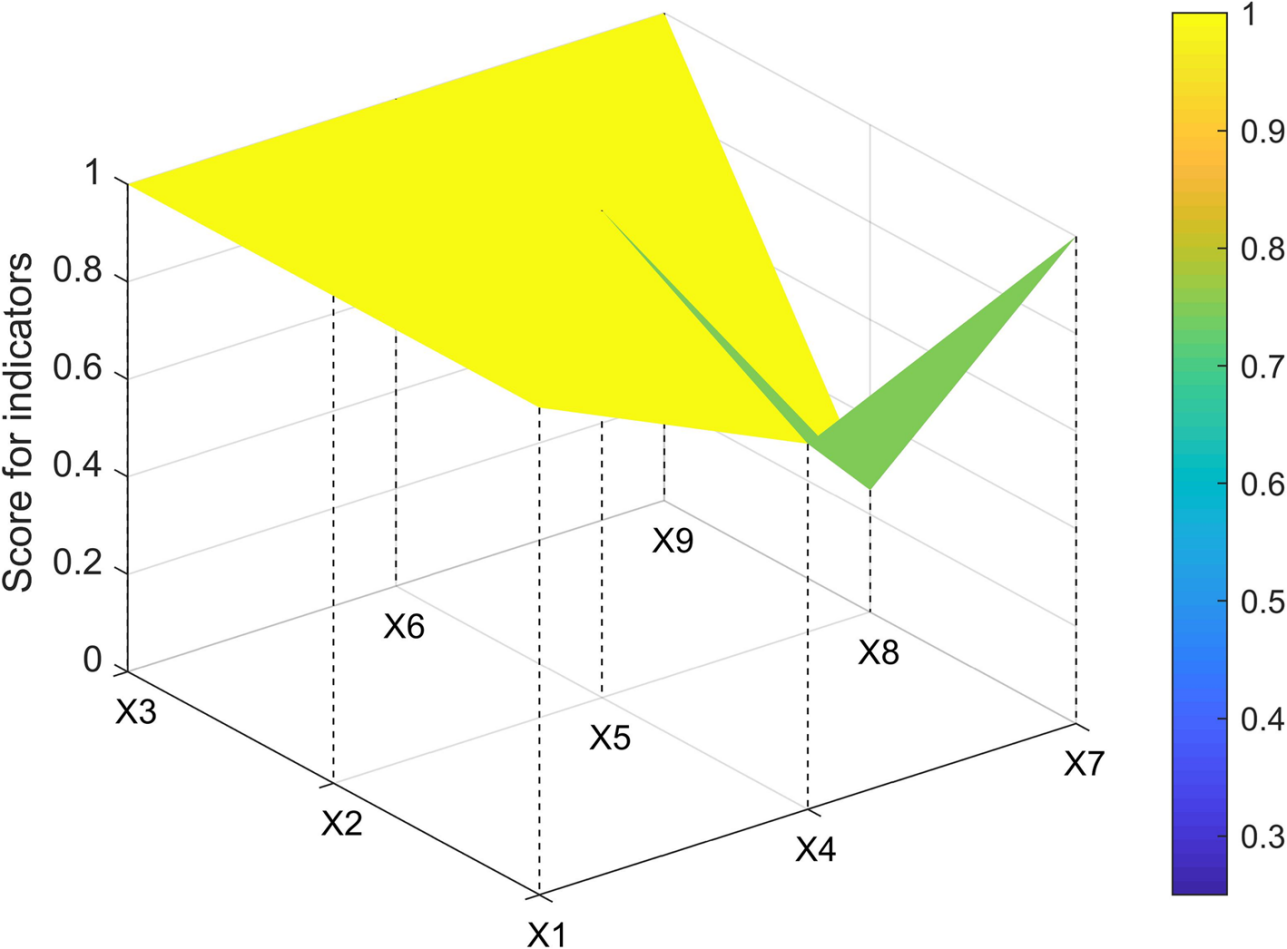
PMC-Surface chart of P7 (Perfect)

The P7 showed a noticeable dent in the X4(incentives and constraints) and X8(issuing agency), with low scores for incentive restraint and release mechanisms (see Fig. 1).

**Fig. 2:**
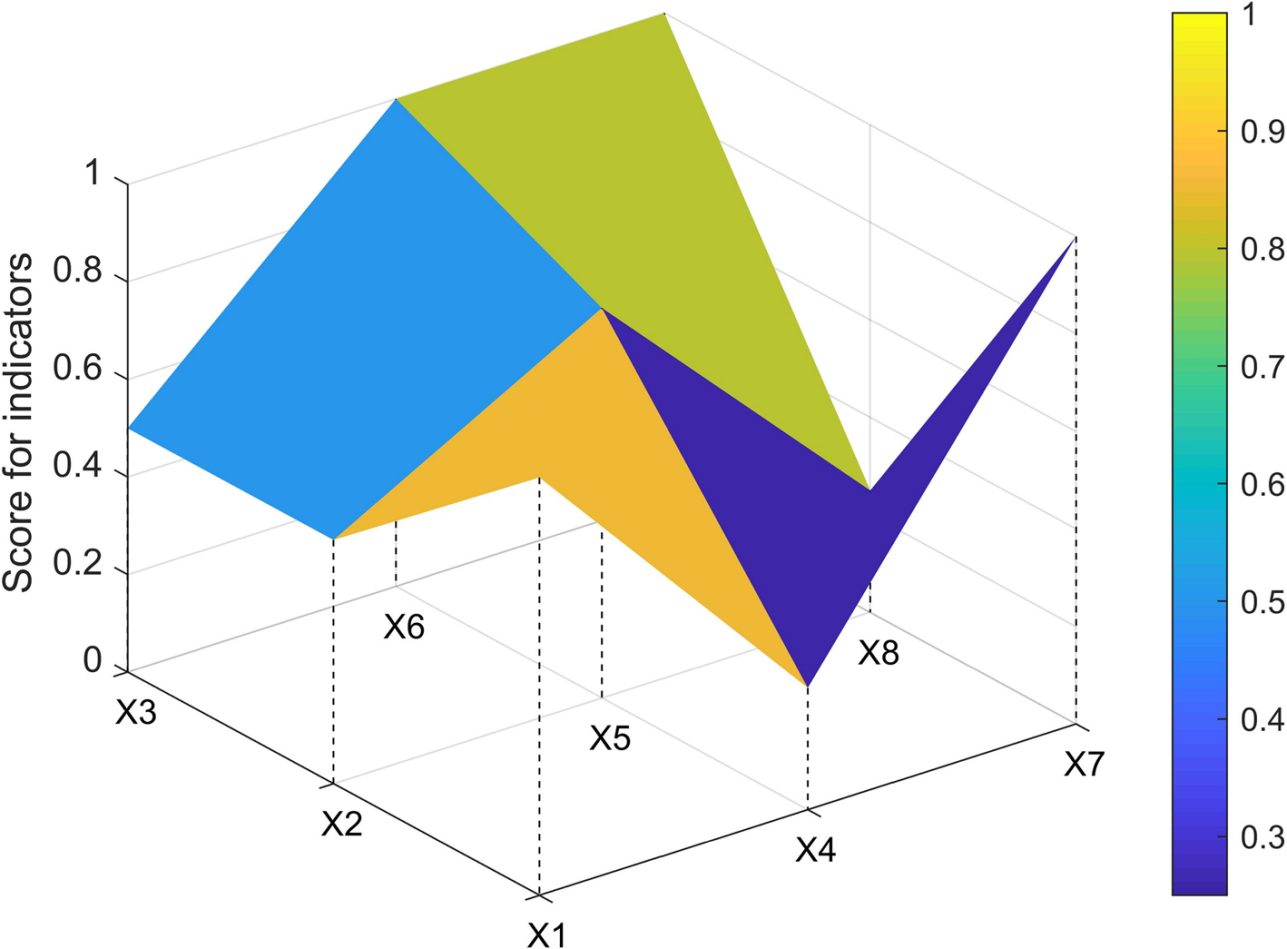
PMC-Surface chart of P1 (Good)

The P1 sagged the most in the X4(incentives and constraints) and X8(issuing authority), followed by the X2(policy timeliness) and X3(policy relevance) (see Fig. 2).

**Fig. 3:**
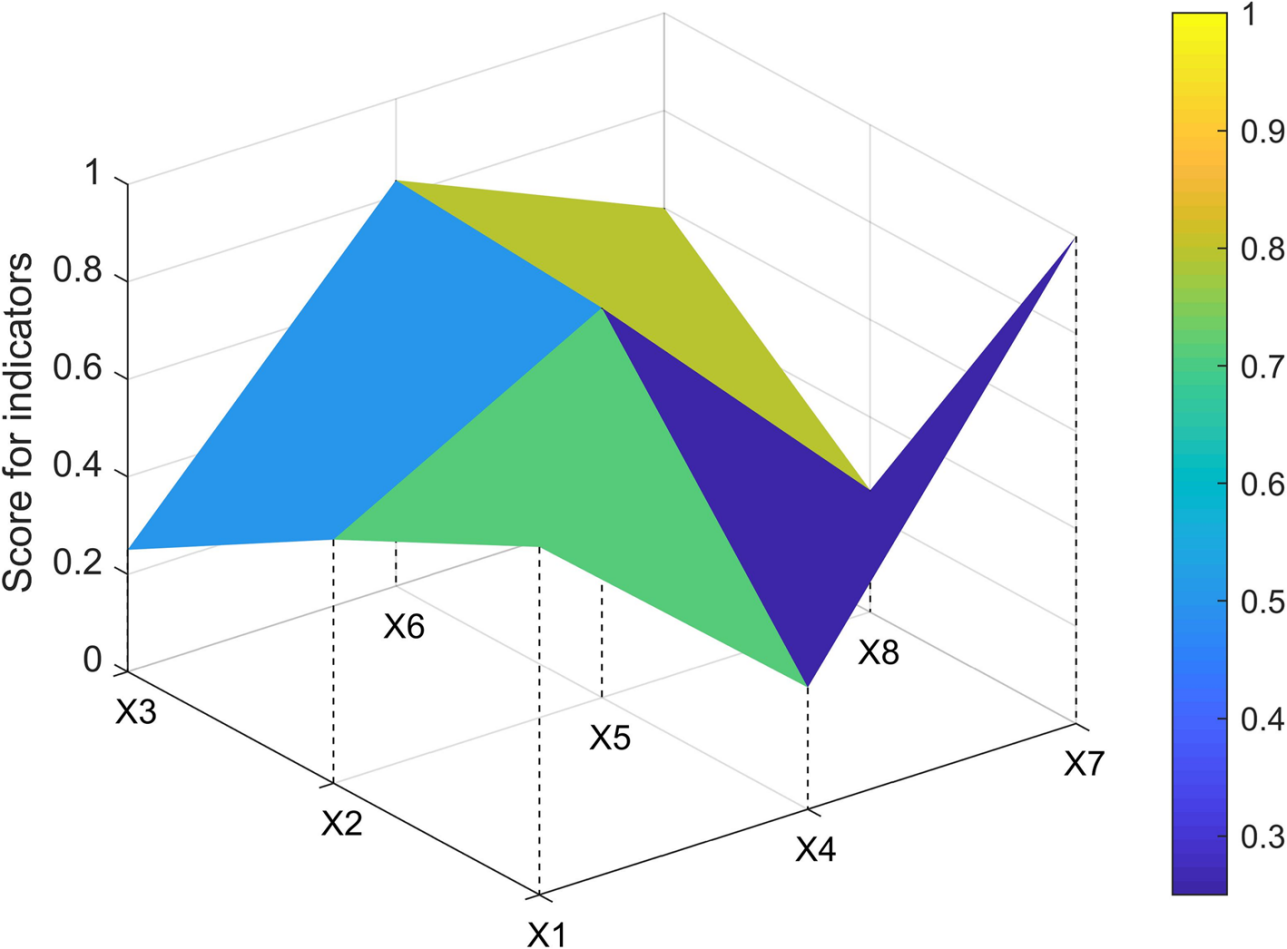
PMC-Surface chart of P3 (Acceptable)

The P3 was dented in all dimensions, with X3(policy relevance) and X4(incentives and constraints) having the most significant impact on curved depressions (see Fig. 3).

**Fig. 4:**
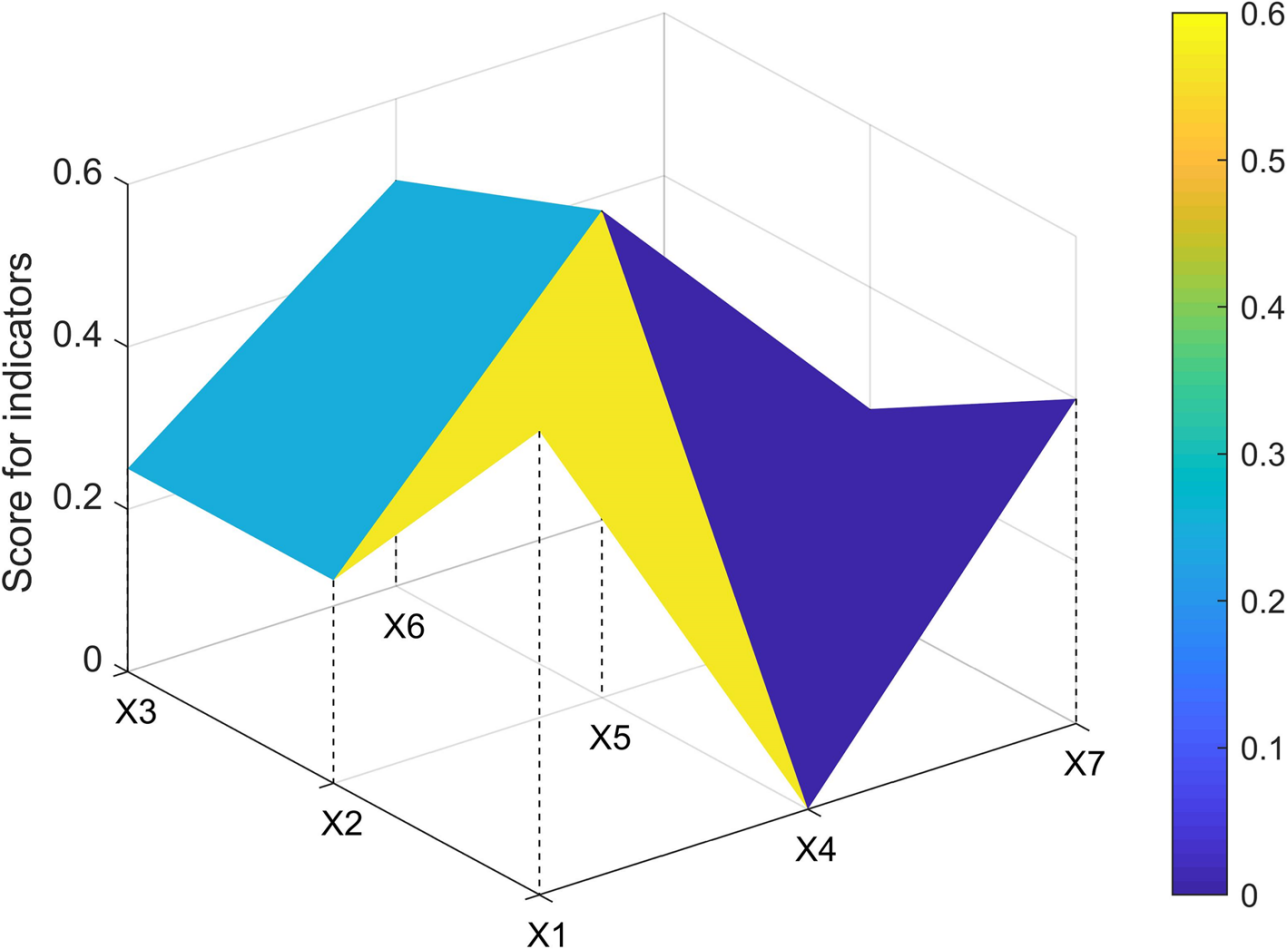
PMC-Surface chart of P33 (Poor)

The P33 had the most dented surfaces, especially on the X4(incentives and constraints), where it scores zero points (see Fig. 4).

#### Comparison between P1 and P3

Based on the analysis above, most policies rank B or C**,** and policies in rank B and policies in rank C differ significantly mainly in the following dimensions: X2 (policy timeliness), X3 (policy relevance), X4 (incentives and constraints), X6 (policy content), X9 (policy objects) (see Table 6). To shed more light on the difference between B-level and C-level policies, we will compare P1 (representing B) with P3 (representing C) in some aspects.

**Table 6:** Comparison Between Rank B and Rank C

**Primary Variables**	**Average of Rank B**	**Average of Rank C**	**Difference**
X1	0.739	0.730	0.009
X2	0.809	0.375	0.434
X3	0.721	0.375	0.346
X4	0.544	0.292	0.252
X5	0.800	0.744	0.056
X6	1.000	0.935	0.065
X7	1.000	0.856	0.144
X8	0.250	0.264	-0.014
X9	1.000	0.856	0.144
X10	1.000	1.000	0.000
PMC	7.863	6.426	1.437

Policy timeliness: P1 scored higher than P3 in policy timeliness. Policy timeliness refers to the time length of policy implementation, which is an essential factor affecting the effect of policy implementation [38]. HPPs are a basic national policy in our country, indicating that it should be implemented and promoted over a long period. Specifically, as implementing health promotion district and county policies requires cross-sectoral collaboration, it takes sufficient time to set up specialized organizations and internal collaboration [39]. The policies of health promotion districts and counties involve creating healthy places, promoting a healthy culture, cultivating healthy people, and creating a healthy environment. With enough time, it can be proved that culture is deeply rooted in the hearts of the people, and the formation of healthy consciousness and habits can be promoted so as to maintain the health and friendly living environment for people.

Policy relevance: P1 scored higher than P3 in policy relevance. Policies not only concern the continuity of time but also the unity and inheritance between policies. Policy relevance is implemented through interpreting the policy content from the perspectives of breadth and depth, to identify the level of government policy formulation stance, and constantly achieve the transition from the guiding ideology to the implementation of the program [40]. Suppose the government does not strengthen the interpretation of relevant documents at all levels in the policy formulation process. In that case, there may be a shortage of policy basis, it may be challenging to get the support of the higher level of government, and there may be a contradiction between the policies.

Policy content: P1 scored higher than P3 in policy content. Based on the available physiological-psychological-social theory, many factors affect health [41]. Therefore, health promotion policies should be formulated comprehensively. We should give concern about system development and organization management, and strengthen the construction of public healthy places and a healthy environment. In addition, we should make detailed plans to spread a healthy culture and foster healthy citizens. Only by ensuring the joint efforts of each component in the policy implementation can the health promotion level of the districts and counties be improved as a whole.

Policy objects: P1 scored higher than P3 in the policy objects. From the ecological perspective, the health level of an area is assessed on all groups, so the layout of HPPs should be carried out from a more macro perspective, including enterprises, institutions, public environment, communities, families, and individuals mentioned in this study [42]. HPPs for enterprises and institutions will be radiated to the healthy transformation of various industries. Considering the construction of the healthy public environment is conducive to providing environmental protection for the physical and mental health of the residents. Community, family, and individual HPPs are the last mile and the final destination for improving the health of the residents.

### Expert field evaluations

Naming each pilot region using Arabic numerals. The name of the pilot area corresponds to the name of the policy. For example, the policy issued by pilot area 1 is P1 (see Table 7).

**Table 7:** Expert Field Evaluations

**Pilot area code**	**1**	**2**	**3**	**4**	**5**	**6**	**7**	**8**	**9**	**10**	**11**	**12**	**13**	**14**	**15**	**16**	**17**	**18**	**19**
Total score	87.66	92.33	84.55	91.59	95.42	77.59	94.80	88.03	90.32	84.06	88.32	90.33	90.59	93.15	88.83	87.36	88.75	93.58	91.07

**Pilot area code**	**20**	**21**	**22**	**23**	**24**	**25**	**26**	**27**	**28**	**29**	**30**	**31**	**32**	**33**	**34**	**35**	**36**	**37**
Total score	91.72	89.68	88.31	85.54	78.66	93.33	86.89	89.30	80.15	86.58	78.84	95.85	81.25	77.94	97.94	78.33	90.85	88.76

### Correlation analysis

**Fig. 5:**
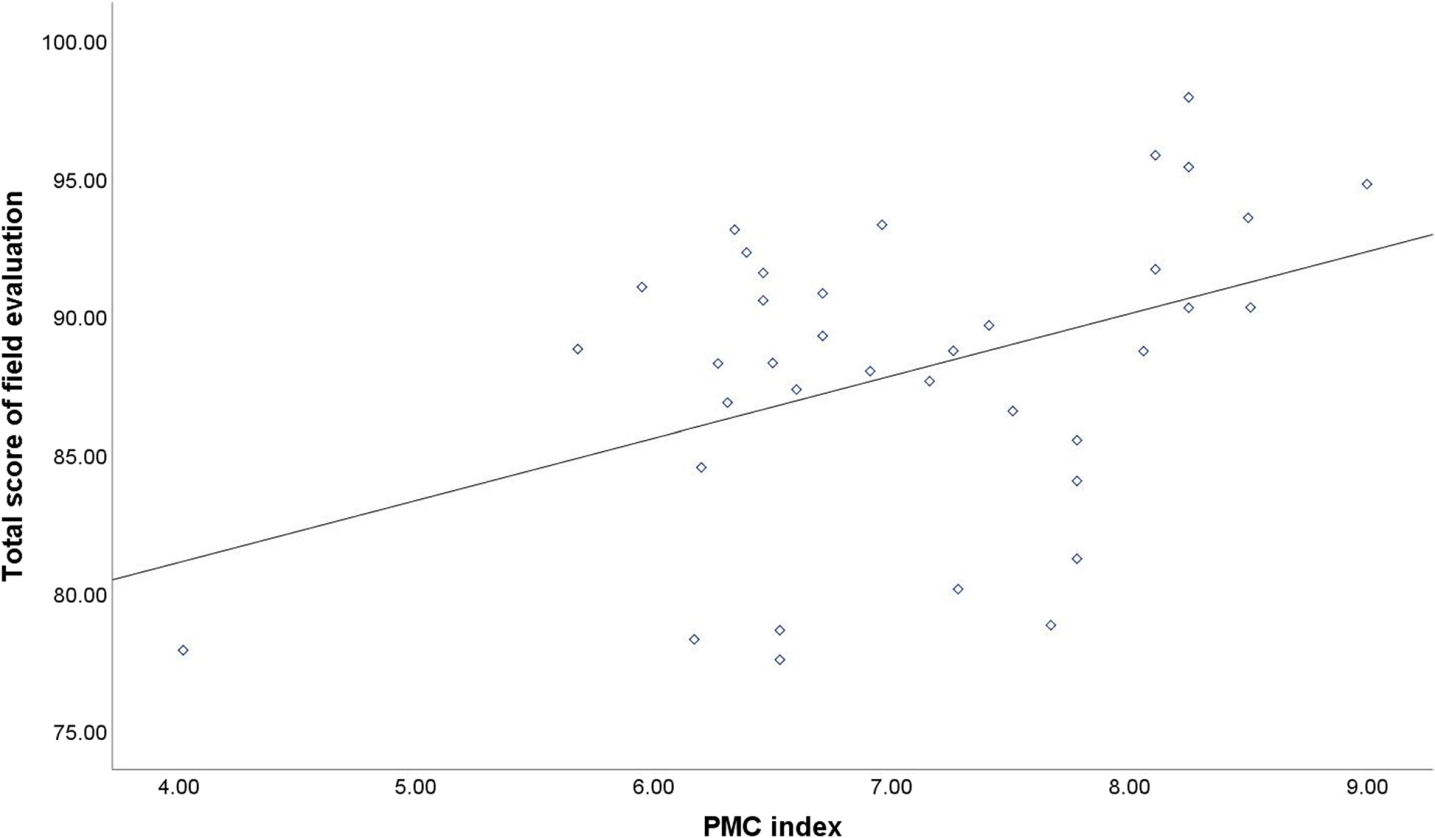
The Relationship Between PMC Index and Total Score of Field Evaluation

The PMC-Index score of the variable and the total score of field evaluation by experts both were continuous variables undergoing the normality test. After correlation analysis, *r*=0.415 and *P*= 0.011 (*P* < 0.05) were obtained, from which it could be concluded that there was a significant correlation between the PMC-Index score and the total score of field evaluation by experts (see Fig. 5).

## Discussion

### The overall evaluation of HPPs

The policy is the guidance of actions [43]. When analyzing HiAP's practical logic, some researchers pointed out that "health first" was the premise in politics [44], and it’s the practical way to formulate health promotion policies from the central government to local governments, targeting at realizing the "Healthy China 2030" strategy. Quantitative evaluation on local health promotion policies is helpful to evaluate whether the influence of policies extends to the grass-roots level and provides scientific basis for deepening health promotion policy reform.

As a strong province of population, economy and culture in western China, Sichuan Province is a demonstration site of healthy culture construction. Therefore, the evaluation of HPPs in this region has important reference value for the formulation of national HPPs. In this study, the health promotion policies of 37 districts (counties) were quantified and evaluated. Overall, the average score of the PMC index was 7.091, indicating that policy consistency was good, and more than 90% of the policy consistency was at a reasonable and acceptable level. However, there is still a gap between policy consistency from these policies and excellent consistency, which shows that there exist some flaws in the process of local policy formulation. In other relevant studies, the PMC index of most studies is acceptable consistency or good consistency, and the results of this study are consistent with those of other studies. This may be related to the fact that local policies are detailed implementation rules by following central policies and local conditions.

### The main factors affecting PMC index score

According to the evaluation dimensions, X2 (policy timeliness), X3 (policy relevance), X4 (incentives and constraints), and X8 (publishing agency) mainly affect the PMC index score.

Most 37 sample policies are temporary and short-term (P1, P2, P3, etc.). However, such health threats as obesity, smoking, diabetes, air pollution, etc., require long-term policy guidance and prevention to make a difference [45]. In addition, some researchers pointed out that the continuity of policies is the task requirement of policy implementation in the new era, and local policies should actively cooperate with the central policies. "Healthy China 2030" is a comprehensive policy covering the short, medium, and long term, so local governments must strengthen the formulation of long-term health promotion policies [36].

The policy relevance can reflect the local government's priority to the superior policy and whether the local policy formulation has a scientific basis. In this study, most sample policies had the best correlation with national-level policies and the worst correlation with municipal-level policies. Some researchers analyzed that the policy is a top-down hierarchical diffusion model many times [46], and the specific policy-making path is meta-policy-basic policy-specific. The districts (counties) strengthen the correlation with municipal-level policies, which is conducive to ensuring the scientificity of the policies and building an excellent political alliance, so as to improve the probability of policy success [47].

The 37 sample policies included in this study seldom involve incentives. From the perspective of policy instruments, appropriate, effective, and incentive policies can promote political stability and economic development [48, 49], but they lack supply-oriented and environmental policy tools in the study. Therefore, in order to ensure the smooth implementation of the health promotion programme, it is necessary to formulate tax relief, administrative approval priority, talent incentives, financial incentives, etc., to motivate all units to participate in health promotion activities and expand the scope of health promotion [50].

From the perspective of publishing agencies, 37 sample policies are all published by government offices, without multi-department joint publication of policies. However, many studies have proved that cross-department collaboration is crucial in promoting HiAP, and the same is valid for health promotion areas [51]. In the process of policy promotion, the health sector cannot be neglected. In fact it plays a leading role, so all relevant departments should actively interact and cooperate [52]. Moreover, the health sector should seek and create shared interests with relevant departments to establish a platform to solve problems [53]. In the future, all districts (counties) should establish a cooperative working mechanism: "government is responsible for leading, departments are responsible for cooperation, and industries are responsible for implementation".

### A significant correlation between policy formulation and implementation

According to the correlation analysis, there is a significant correlation between the PMC index score and the total score of field evaluation, which indicates a correlation between policy formulation and implementation, and proves the importance of establishing an integrated mechanism for policy formulation and implementation [54]. For example, the personnel involved in implementing any policy must have sufficient knowledge of the policy, even those involved in policy implementation must also be involved in the process of policy formulation.

Through the analysis of policy consistency and the correlation between policy formulation and policy implementation, this study theoretically provides a reference basis for HPPs policy formulation and enriches the evaluation research of HPPs. In fact, integrating health into all policies is an important and complex project, which refers to integrating the three levels of government, society and individuals to form a strong synergy to maintain and promote health [55]. In policy-making, local governments should follow the template of policies, strengthen departmental collaboration, and promote policy cohesion. In policy practice, all industries in the society follow HPPs related rules and regulations to promote the horizontal and vertical development of HPPs. At the same time, through various forms, such as publicity, cultural activities, increase of sports equipment and facilities, it is advocated to integrate HPPs into personal life, to promote the health of the whole society.

### Limitations

However, the policies selected in this study may have some limitations due to policy openness and website construction, such as insufficient representation. In the future, we can optimize the problem by cooperating with government departments or using crawler mining to achieve more objective and scientific policy evaluation.

## Conclusions

Based on PMC - index model, HPPs evaluation system related to the counties/districts of Sichuan province is established in this study through conducting the quantitative analysis of HPPs in the counties/districts of Sichuan province, and combining the expert field evaluations, which explored the Sichuan HPPs consistency as well as the correlation of policy formulation and implementation, for the continuous reform of HPPs to provide theoretical reference.

## Data Availability

The datasets used and analysed during the current study are available from the corresponding author on reasonable request.

## Abbreviations

HPPs: Health Promotion Policies
HiAP: Health in All Policies
HPPsC: Health Promotion Policies Criteria

## References

[1] Synnevag ES, Amdam R, Fosse E (2018). Intersectoral Planning for Public Health: Dilemmas and Challenges. Int J Health Policy Manage..

[2] Gase L, Kuo T, Teutsch S, Fielding J (2014). Estimating the Costs and Benefits of Providing Free Public Transit Passes to Students in Los Angeles County: Lessons Learned in Applying a Health Lens to Decision-Making. Int J Environ Res Public Health..

[3] Maigeng Z, Haidong W, Xinying Z, Peng Y, Jun Z, Wanqing C (2020). Mortality, morbidity, and risk factors in China and its provinces, 1990–2017: a systematic analysis for the Global Burden of Disease Study 2017. Lancet..

[4] Safi M, Bertram ML, Gulis G (2020). Assessing Delivery of Selected Public Health Operations via Essential Public Health Operation Framework. Int J Env Res Pub He..

[5] Ooms G, Kruja K (2019). The integration of the global HIV/AIDS response into universal health coverage: desirable, perhaps possible, but far from easy. Globalization Health..

[6] Zhang KL, Ran B (2022). Active Health Governance-A Conceptual Framework Based on a Narrative Literature Review. Int J Env Res Pub He..

[7] Van Vliet-Brown CE, Shahram S, Oelke ND (2018). Health in All Policies utilization by municipal governments: scoping review. Health Promot Int..

[8] Yanfei Y, Lin W, Hongwei X, Jieyan G (2015). Integrate health into all policy theory and international experience. Health Education in China..

[9] Peters D, Harting J, van Oers H, Schuit J, de Vries N, Stronks K (2016). Manifestations of integrated public health policy in Dutch municipalities. Health Promot Int..

[10] Pinto AD, Molnar A, Shankardass K, O'Campo PJ, Bayoumi AM (2015). Economic considerations and health in all policies initiatives: evidence from interviews with key informants in Sweden, Quebec and South Australia. BMC Public Health..

[11] Hofstad Hege (2016). The ambition of Health in All Policies in Norway: The role of political leadership and bureaucratic change. Health Policy..

[12] Zeeb H, Hilderink H, Forberger S (2018). Bundesgesundheitsblatt Gesundheitsforschung Gesundheitsschutz..

[13] Newman L, Ludford I, Williams C, Herriot M (2016). Applying Health in All Policies to obesity in South Australia. Health Promot Int..

[14] Qichao C, Shi-rong L (2015). Integrate health into the implementation of all policies in the construction of health promoting hospitals. Health Educ China..

[15] Vassiliou AG, Georgakopoulou C, Papageorgiou A, Georgakopoulos S, Goulas S, Paschalis T (2020). Health in All Policy Making Utilizing Big Data. Acta Inform Med..

[16] Steenbakkers M, Jansen M, Maarse H, de Vries N (2012). Challenging Health in All Policies, an action research study in Dutch municipalities. Health Policy..

[17] Arredondo Malagon, Curl Corburn (2014). Health in all Urban policy: City services through the prism of health. J Urban Health: Bull New York Acad Med..

[18] Ketan S, Emilie R, Carles M, Patricia O (2014). Strengthening the implementation of Health in All Policies: A methodology for realist explanatory case studies. Health Policy Plann..

[19] Moses MW, Pedroza P, Baral R, Bloom S, Brown J, Chapin A (2018). Funding and services needed to achieve universal health coverage: applications of global, regional, and national estimates of utilisation of outpatient visits and inpatient admissions from 1990 to 2016, and unit costs from 1995 to 2016. Lancet Public Health..

[20] Lu C, Jin S, Tang X, Lu C, Pang J (2020). Spatio-Temporal Comprehensive Measurements of Chinese Citizens' Health Levels and Associated Influencing Factors. Healthcare..

[21] Cai P, Wu X, Liu Z, Deng Y, Chen X, Yi G (2019). Analysis of the burden and trend of injury in Sichuan, China, from 2006 to 2015: results from the national injury surveillance system. BMJ Open..

[22] Akhnif EH, Hachri H, Belmadani A, Mataria A, Bigdeli M (2020). Policy dialogue and participation: a new way of crafting a national health financing strategy in Morocco. Health Res Policy Sy..

[23] Mbachu CO, Onwujekwe O, Chikezie I, Ezumah N, Das M, Uzochukwu B (2016). Analysing key influences over actors' use of evidence in developing policies and strategies in Nigeria: a retrospective study of the Integrated Maternal Newborn and Child Health strategy. Health Res Policy Sy..

[24] Estrada M (2011). Policy modeling: Definition, classification and evaluation. J Policy Model..

[25] Dai SL, Zhang WM, Zong JM, Wang YY, Wang G (2021). How Effective Is the Green Development Policy of China's Yangtze River Economic Belt? A Quantitative Evaluation Based on the PMC-Index Model. Int J Env Res Pub He..

[26] Feng H, Xiaoni Q, Xiaoyan W (2020). Quantitative evaluation of robot industrial Policy based on PMC Index model: A case study of 8 robot industrial policy information. J Inform..

[27] Liying X, Han Q, Li X, Shuzhen C (2020). Research on Policy Evaluation of biomedical Industry based on PMC Index. Chin J New Med..

[28] Yating L, Reddick (2021). Evaluation and Optimization of water resources pollution control Policies for high-quality green Development: Based on PMC Index model. Res Industries..

[29] Haiwei Z, Lianghui F (2021). Research on Quantitative evaluation and optimization Path of reservoir migration policy based on PMC index model. Water Conservancy Econ..

[30] Tingting J, Guofang M, Guixiang H, Water HI (2021). Policy analysis and Quantitative evaluation of the combination of medical and nursing care in China: Based on PMC index model. Soft Sci Health..

[31] Siqi Z, Dongliang L, Yuqi X, Shuzhen C (2021). Quantitative evaluation of chronic disease Management Policy based on PMC Index model. Chinese Pharm..

[32] Yuying Z, Zhengrong L (2020). Quantitative Evaluation of central old-age service policy content: Based on PMC Index model. J Univ Electron Sci Technol China..

[33] Ji Y. Research on Quantitative Evaluation of farmland transfer Policy based on PMC index model: Tianjin Polytechnic University; 2021.

[34] Wenjing Z, Li Z, Jun Y (2021). Long-term care insurance policy Evaluation: Based on PMC Index model. Manage Health Serv China..

[35] Jinming X, Baojun Z (2022). Quantitative evaluation of Chinese sports Education Integration Policy based on PMC-AE index model. J Phys Educ..

[36] Yang J, Siri JG, Remais JV, Cheng Q, Zhang H, Chan K (2018). The Tsinghua-Lancet Commission on Healthy Cities in China: unlocking the power of cities for a healthy China. Lancet..

[37] Kuang B, Han J, Lu X, Zhang X, Fan X (2020). Quantitative evaluation of China's cultivated land protection policies based on the PMC-Index model. Land Use Policy..

[38] Mwendera CA, De Jager C, Longwe H, Kumwenda S, Hongoro C, Phiri K (2019). Challenges to the implementation of malaria policies in Malawi. BMC Health Serv Res..

[39] van Eyk H, Harris E, Baum F, Delany-Crowe T, Lawless A, MacDougall C (2017). Health in All Policies in South Australia-Did It Promote and Enact an Equity Perspective?. Int J Env Res Pub He..

[40] Jin Y, Xu J, Zhu W, Zhang Y, Meng Q (2020). Synergy of policies to strengthen primary care: evidence from a national repeated cross-sectional study. BioMed Central..

[41] Jin P, Gao YS, Liu LB, Peng ZH, Wu H (2019). Maternal Health and Green Spaces in China: A Longitudinal Analysis of MMR Based on Spatial Panel Model. Healthcare..

[42] Cometto G, Nartey E, Zapata T, Kanda M, Md Y, Narayan K (2019). Analysing public sector institutional capacity for health workforce governance in the South-East Asia region of WHO. Hum Resour Health..

[43] Gilmour L, Duncan E, Maxwell M. Policy addressing suicidality in children and young people: a scoping review protocol. BMJ Open. 2018;8(9):e23153. 10.1136/bmjopen-2018-023153 PMC6144395 30224396

[44] Shankardass K, Muntaner C, Kokkinen L, Shahidi FV, Freiler A, Oneka G (2018). The implementation of Health in All Policies initiatives: a systems framework for government action. Health Res Policy Syst..

[45] Caplette ME, Provencher V, Bissonnette-Maheux V, Dugrenier M, Lapointe A, Gagnon MP (2017). Increasing Fruit and Vegetable Consumption Through a Healthy Eating Blog: A Feasibility Study. JMIR Res Protocols..

[46] Goodman C, Kachur SP, Abdulla S, Bloland P, Mills A (2007). Drug shop regulation and malaria treatment in Tanzaniawhy do shops break the rules, and does it matter. Health Policy Plann..

[47] Mwendera C, De Jager C, Longwe H, Hongoro C, Phiri K, Mutero CM (2017). Development of a framework to improve the utilisation of malaria research for policy development in Malawi. Health Res Policy Syst..

[48] Tambor M, Pavlova M, Golinowska S, Arsenijevic J, Groot W (2016). Financial incentives for a healthy life style and disease prevention among older people: a systematic literature review. BMC Health Serv Res..

[49] Kahn JG, Harris B, Mermin JH, Clasen T, Lugada E, Grabowksy M (2011). Cost of Community Integrated Prevention Campaign for Malaria, HIV, and Diarrhea in Rural Kenya. BMC Health Serv Res..

[50] Yamashita R, Sato S, Akase R, Doi T, Harada E (2021). Effects of social network incentives and financial incentives on physical activity and social capital among older women: a randomized controlled trial. BMC Public Health..

[51] Huang NC, Kuo HW, Hung TJ, Hu SC (2019). Do Healthy City Performance Awards Lead to Health in All Policies? A Case of Taiwan. Int J Env Res Pub He..

[52] Jabot F, Tremblay E, Rivadeneyra A, Diallo T, Lapointe G (2020). A Comparative Analysis of Health Impact Assessment Implementation Models in the Regions of Montérégie (Québec, Canada) and Nouvelle-Aquitaine (France). Multidisciplinary Digital Publishing Inst..

[53] Ashraf S, Moore C, Gupta V, Chowdhury A, Azad AK, Singh N, et al. Overview of a multi-stakeholder dialogue around Shared Services for Health: the Digital Health Opportunity in Bangladesh. Health Res Policy Syst. 2015;13:74. 10.1186/s12961-015-0063-2. 10.1186/s12961-015-0063-2 PMC4673719 26646372

[54] Banchani E, Tenkorang EY (2014). Implementation challenges of maternal health care in Ghana: the case of health care providers in the Tamale Metropolis. BMC Health Serv Res..

[55] The CPC Central Committee and The State Council issued the Outline of the Healthy China 2030 Plan. Bull State Council People's Republic of China. 2016(32):5–20.

